# 胸部CT肺血管造影辅助诊断侵袭性肺曲霉病：3例临床分析

**DOI:** 10.3760/cma.j.cn121090-20250220-00082

**Published:** 2025-10

**Authors:** 登梅 田, 建华 游, 炯 胡, 苓 王

**Affiliations:** 1 上海交通大学医学院附属瑞金医院血液科，上海 200025 Shanghai Institute of Hematology, State Key Laboratory of Medical Genomics, National Research Center for Translational Medicine at Shanghai, Ruijin Hospital Affiliated to Shanghai Jiao Tong University School of Medicine, Shanghai 200025, China; 2 上海交通大学医学院附属瑞金医院海南医院血液科，海南 460000 Department of Hematology, Ruijin-Hainan Hospital, Shanghai Jiaotong University School of Medicine, Hainan 460000, China

## Abstract

胸部CT肺血管造影（CTPA）对侵袭性肺曲霉病（IPA）临床诊断具有一定价值。本文报道了3例血液系统恶性肿瘤患者（包括异基因造血干细胞移植2例和化疗1例），在接受移植或化疗后，均出现发热且抗生素治疗48 h后未好转，行胸部高分辨CT平扫（HRCT）检查，发现至少一个直径大于10 mm的致密肺实变影，遂行CTPA检查。经CTPA检查发现存在血管阻塞征（VOS）阳性的患者2例，VOS阴性1例，其中2例VOS阳性患者拟诊IPA，予诊断驱动抗真菌治疗后病情好转。1例VOS阴性患者，确诊为弥漫大B细胞淋巴瘤肺部病灶累及，予抗淋巴瘤治疗后，病灶明显缩小。提示CTPA检测到的VOS具有一定特征性，对IPA高危患者，有助于提高影像学诊断的特异性，指导临床治疗决策。

## Abstract (en)

Chest CT pulmonary angiography（CTPA）has certain auxiliary diagnostic value for the clinical diagnosis of invasive pulmonary aspergillosis（IPA）. Three patients with hematological malignancies were reported, including 2 ones after allogeneic hematopoietic stem cell transplantation and 1 ones after chemotherapy for refractory recurrent leukemia. Each patient was treated with antibiotics for at least 48 hours after the onset of fever, they all underwent chest high-resolution CT（HRCT）scans without fever resolution. CT revealed at least one dense pulmonary consolidation shadow with a diameter greater than 10 mm, and subsequently a CTPA examination was performed to observe the effect of CTPA imaging signs for the diagnosis of IPA. There were 2 patients with positive vascular occlusion sign（VOS）and 1 patient with negative VOS detected by CTPA. Among them, 2 patients with positive VOS were diagnosed with possible IPA and received with diagnosis-driven antifungal treatment, which improved their conditions. One patient with negative VOS sign was diagnosed with diffuse large B-cell lymphoma involving the lungs. After receiving anti-lymphoma treatment, the lesions significantly reduced in size. The vascular occlusion sign detected by CTPA is relatively characteristic. For high-risk IPA patients, it helps to improve the specificity of imaging diagnosis and guide clinical treatment decisions.

侵袭性肺曲霉病（Invasive pulmonary aspergillosis, IPA）是血液系统恶性肿瘤患者治疗过程中可能发生的严重感染并发症[1]–[2]。由于病原菌培养方法敏感性差，CT影像学表现缺乏特异性，IPA早期诊断困难[3]–[4]。2020年欧洲癌症治疗和研究组织和真菌病研究组（EORTC/MSG）共识对IPA的CT影像学标准进行了更新，新增了楔形和节段性或大叶性实变为IPA影像学的特异性表现[5]。尽管其敏感性高，但相对特异性较差[6]。

血管阻塞征（Vessel occlusion sign，VOS）是胸部CT肺血管造影（Computed tomography pulmonary angiogram，CTPA）诊断IPA的重要影像学特征，其病理基础为真菌菌丝阻塞血管或侵袭血管壁，在CTPA影像上表现为血管截断或管腔内对比剂充盈缺损的现象[7]。本研究，我们通过对3例恶性血液病伴肺部阴影的患者进行CTPA检查，初步评估VOS在IPA影像学表现的辅助诊断价值。

## 病例资料

例1，男，60岁，难治性急性髓系白血病（AML），采用CLAG-E大剂量化疗（G-CSF+阿糖胞苷+克拉屈滨+依托泊苷）序贯异基因造血干细胞移植（allo-HSCT）（子供父，HLA 5/10相合）进行治疗，氟达拉滨+白消安（Flu-Bu）为预处理方案，后置环磷酰胺+兔抗人胸腺细胞免疫球蛋白+他克莫司（PT-Cy+ATG+TAC）预防移植物抗宿主病（GVHD）。患者呈疾病未缓解状态且预估治疗后中性粒细胞缺乏时间长于10 d，口服泊沙康唑预防侵袭性真菌病（Invasive fungal disease，IFD）。前期减瘤化疗过程中出现中性粒细胞缺乏伴发热，血液细菌及真菌培养均阴性，给予亚胺培南西司他丁钠经验性治疗后好转。–2 d患者再次出现发热，T_max_ 38.9 °C，伴畏寒寒战、口腔黏膜炎、呕吐，无咳嗽咳痰等症状，C反应蛋白（CRP）48.3 mg/L，降钙素原（PCT）18.63 ng/ml，血清（1,3）-β-D葡聚糖试验（G试验）和半乳甘露聚糖试验（GM试验）均阴性，多次咽拭子培养均未见细菌或真菌生长。停用亚胺培南西司他丁钠，予替加环素联合阿米卡星及利奈唑胺治疗7 d，发热仍未好转。+6 d行胸部CT示左肺上叶局部实变（图1A），+7 d行CTPA显示：左肺上叶局部实变区血管中断（图1B、1C），即VOS阳性，拟诊IPA，予伏立康唑+卡泊芬净两联抗真菌治疗。抗真菌治疗1个月后复查CTPA，左上肺实变明显缩小（图1D）。持续抗真菌药物治疗7周后复查胸部CT，病灶体积显著缩小。停用卡泊芬净，继续口服伏立康唑（每12 h 200 mg）出院后维持治疗，抗真菌治疗11周后复查胸部CT示左上肺实变影进一步缩小（图1E）。

**图1:**
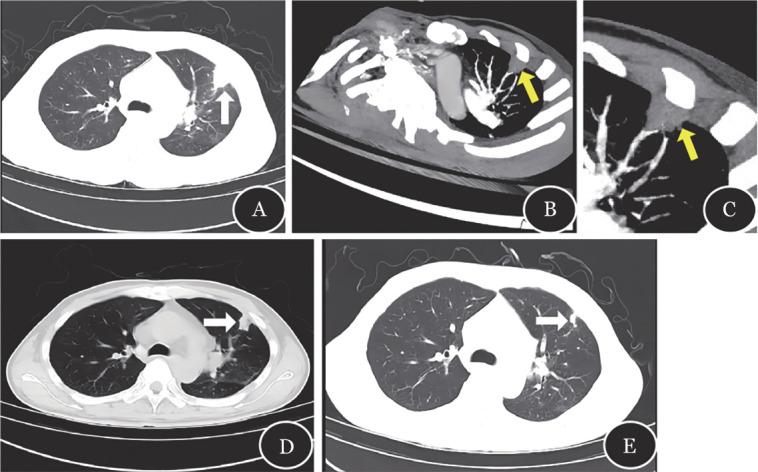
例1的CT及CT肺血管造影的影像学表现 **A** 左肺上叶局部实变（白色箭头示）；**B** 左肺上叶局部实变区血管中断（黄色箭头标示血管阻塞征阳性）；**C** 图B的局部放大；**D** 抗真菌治疗1个月后，左上肺实变明显缩小；**E** 抗真菌治疗11周后，左上肺实变影进一步缩小

例2，男，29岁，AML（FLT3-ITD突变），化疗后复发。CLAG-E方案大剂量化疗序贯allo-HSCT。Flu-Bu方案预处理，PT-Cy+ATG+TAC预防GVHD，氟康唑预防IFD。+1 d出现发热，T_max_ 39 °C，伴畏寒，无咳嗽胸痛等，CRP 89 mg/L，PCT（–），G/GM（–），血培养（–），其他病原体筛查均无阳性发现，先后予头孢哌酮-舒巴坦钠、美罗培南、利奈唑胺静脉抗感染，治疗有效后减停，体温正常。+18 d粒系植入，+21 d巨核系植入。+21 d再次发热，伴右侧胸痛、咳嗽、咳少许白痰，行痰液细菌培养：可见绿色链球菌和干燥奈瑟菌等呼吸道正常菌群定植，痰液真菌培养阴性，痰液免疫荧光染色未见真菌。肺部CT及CTPA检查示：右肺下叶背段节段实变影（图2A）；右肺下叶基底段动脉局部充盈缺损，VOS（+）（图2B、2C），拟诊IPA，将氟康唑调整为伏立康唑抗真菌治疗，并予抗凝治疗，体温正常。伏立康唑治疗约2周复查CT示右肺下叶背段病变较前缩小，病变空洞形成伴结节影，新月征，考虑抗曲霉治疗后变化（图2D）。伏立康唑治疗约5周，胸部CT示病灶内空洞较前增大、实性成分较前缩小，考虑曲霉菌感染治疗有效。伏立康唑治疗15周后，复查CT及CTPA，右肺下叶背段病灶较前范围缩小，腔内空洞、积液吸收（图2E）；再次肺动脉CT血管造影未见明显异常，VOS消失（图2F）。

**图2:**
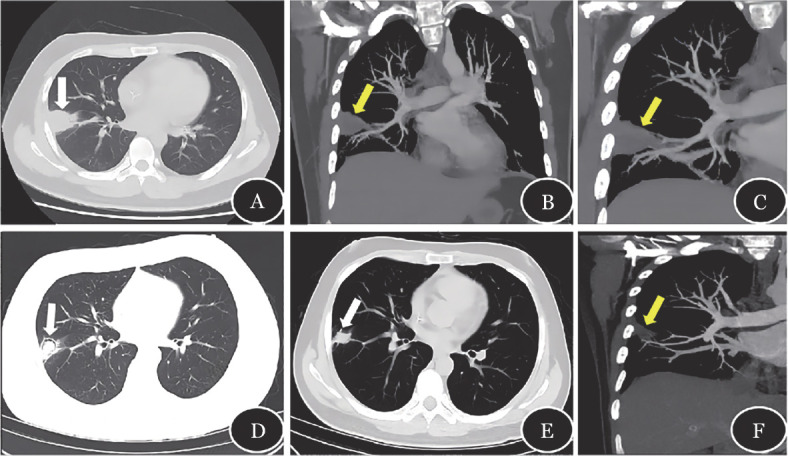
例2的CT及CT肺血管造影的影像学表现 **A** 右肺下叶背段节段实变影（白色箭头示）；**B** 右肺下叶基底段动脉局部充盈缺损（黄色箭头示血管阻塞征阳性）；**C** 图B的局部放大；**D** 伏立康唑治疗约2周，右肺下叶背段病变较前缩小，病变空洞形成伴结节影；**E** 伏立康唑治疗15周后，右肺下叶背段病灶较前片范围缩小，腔内空洞、积液吸收；**F** 伏立康唑治疗15周后，肺动脉CT血管造影未见明显异常，血管阻塞征消失（黄色箭头示血管阻塞征阴性）

例3，男，22岁，Ph^+^急性淋巴细胞白血病（Ph^+^ALL），单倍体造血干细胞移植（haplo-HSCT）继发植入失败，行HLA 8/10相合无关供者来源的二次移植。予Flu-Bu-ATG方案预处理后回输外周血造血干细胞，环孢素A（CsA）+吗替麦考酚酯（MMF）+甲氨蝶呤（MTX）预防GVHD。+12 d巨核系植入，+13 d粒系植入。卡泊芬净预防IFD。+33 d患者出现高热、咳嗽、咳少许痰，中性粒细胞已植入，CRP 85.7 mg/L，PCT 0.57 ng/ml，G/GM（–），CMV-DNA 5.38×10^4^拷贝/ml，EBV-DNA 2.7×10^5^拷贝/ml且在之后的一周内持续增长至4.1×10^6^拷贝/ml。痰液细菌培养可见绿色链球菌和干燥奈瑟菌等呼吸道正常菌群定植，痰液真菌培养阴性，呼吸道常见病毒抗体及核酸检测均阴性。予美罗培南+利奈唑胺经验性抗感染、更昔洛韦抗病毒。+35 d行胸部CT示：两肺多发结节、肿块影（图3A）。考虑IPA可能，加用两性霉素B联合伏立康唑抗真菌治疗，体温无明显好转。+38 d行CTPA检查示：肺动脉干及主要分支未见明确栓塞；两肺内见多发斑片、斑结影及小结节，右肺下叶占位，可见血管完整穿透病灶（图3B、3C）。考虑VOS（–），停用两性霉素B，并行CT引导下肺组织活检。肺穿刺标本病理考虑移植后淋巴组织增殖性疾病，单形性，单克隆，B细胞，合并EBV感染，符合移植后EBV（+）的弥漫性大B细胞淋巴瘤。基因重排检测结果：B淋巴瘤克隆基因重排结果为阳性。予利妥昔单抗治疗4次后，复查胸部CT，右下肺肿块影消失（图3D）。

**图3:**
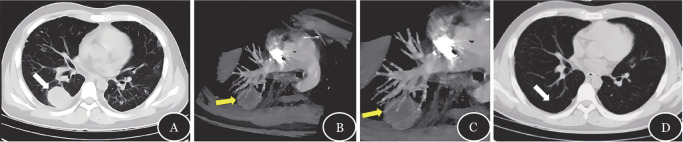
例3的CT及CT肺血管造影的影像学表现 **A** 右下肺肿块影（白色箭头示）；**B** 右下肺的肿块影可见血管完整穿透病灶（黄色箭头示血管阻塞征阴性）；**C** 图B的局部放大；**D** 右下肺肿块影消失

## 讨论及文献复习

IPA是血液系统恶性肿瘤重要的死亡原因之一[1]。国内前瞻性、多中心流行病学研究显示，HSCT病例中确诊和临床诊断IFD患者中，曲霉菌占70.6％，相关死亡率为18.6％[8]。美国移植相关感染监测网络（TRANSNET）显示HSCT后IFD发生率为3.4％，IFD相关死亡率50％以上，1年内IPA相关死亡率为26％[9]。但是目前IPA的确诊仍然具有很大的挑战。根据2020年EORTC/MSG共识定义，只有无菌部位的组织学检查和（或）阳性培养结果才能提供IPA的明确证据。在临床常规中，特别是在标准的预防性或经验性抗真菌治疗中，微生物检测，如培养物甚至GM试验或G试验等生物标志物，往往是阴性的[10]–[11]。没有真菌学阳性证据但临床怀疑IPA的患者被认为是“拟诊IPA”，胸部CT影像学是此类患者的诊断核心。2020年EORTC/MSG共识规定：CT影像学出现伴或不伴晕征（halo sign）的致密边界清楚病变、空气新月征（air-crescent sign）、空洞、楔形/节段性或大叶性实变是临床诊断IPA的影像学标准[5]。Greene等[12]分析了235例IPA患者的胸部CT结果，显示IPA影像表现呈多种改变，94％患者至少有1个大结节（直径大于1 cm），且61％患者伴有晕征。其他影像学表现，包括实变（30％）、梗死样结节（27％）、空洞病变（20％）和空气新月征（10％），则较少见。但大结节、晕征并非IPA胸部CT特有的表现，其他感染性病原体，如假单胞菌和隐球菌，以及肿瘤和自身炎症也可以产生类似CT表现。基于普通CT表现怀疑为IPA的患者中，仅50％患者经组织病理学或尸检结果确诊为IPA[13]。

鉴于CT的重要作用和目前的局限性，如何提高IPA影像学证据的特异性，众多学者做了积极探索。Sonnet等[14]首次利用CTPA检测IPA的血管侵袭，该研究对10例疑似IPA的患者进行CTPA，在组织病理学证实为有真菌血管侵袭的5个病灶中，有4个病灶VOS呈阳性，敏感性为80％。1例肺毛霉病患者显示VOS假阴性。在非真菌病原体侵袭的9个病灶中未显示任何阳性VOS，其特异性为100％。Stanzani等[15]进行了一项单中心、前瞻性、非随机试验，纳入了36例临床怀疑为侵袭性霉菌病（IMD）的血液系统恶性肿瘤患者。研究发现确诊IMD的5例患者CTPA结果均显示病灶边缘血管中断（VOS阳性率100％）；7例临床诊断IMD的患者中有5例显示VOS阳性；24例拟诊IMD的患者中，15例最终排除IMD，其中14例患者的CTPA结果为VOS阴性，1例患者因患有金黄色葡萄球菌脓毒性栓塞致VOS假阳性，其余9例患者最终诊断IMD并显示VOS阳性。根据EORTC/MSG共识的最终诊断与CTPA结果的相关性分析，VOS诊断IMD的敏感性为95％，特异性为87.5％，阳性预测值为90.5％，阴性预测值为93.3％。因而，对伴有明确界限病灶的疑似IMD的患者，CTPA同时兼具较好的诊断及排除诊断价值。

CTPA可以提高CT对IPA的诊断能力，但其相对于其他CT征象（如晕征、低密度征、胸腔积液、反晕征等）的表现尚不清楚。一项意大利的前瞻性临床研究纳入100例疑似IPA的患者，比较CTPA与普通CT对于IPA的诊断价值[16]。研究发现：CTPA敏感性为98％，特异性为89％，阳性似然比（Positive likelihood ratio，PLR）为8.86，阴性似然比（Negative likelihood ratio，NLR）为0.02，诊断优势比（Diagnostic odds ratio，DOR）为406.4。然而，晕征的敏感性为69％，特异性为65％，PLR为2，NLR为0.47，DOR为4.16，VOS的诊断优势比相比晕征高约100倍。这与Henzler等[13]的结论一致。此外，研究还显示根据CTPA的检测结果，VOS阴性的患者静脉或口服抗真菌治疗比阳性患者停止得更早（*P*≤0.001）。因此，VOS是目前血液系统恶性肿瘤患者诊断IPA最敏感、可能也是最特异的影像学征象。VOS阴性也提高了排除IPA的准确性，支持停止抗真菌治疗。

本文中我们的3例患者均具有多项IFA高危因素，给予抗真菌预防治疗，在患者发热过程中，病原微生物的培养及G/GM试验均为阴性，这与抗真菌治疗会显著降低血清G/GM试验及微生物培养的敏感性的研究一致。其他的检测手段如PCR，由于多数单位没有实现标准化，因而尚未在临床实践中常规应用，二代测序（NGS）因其超高敏感性带来背景混杂或污染病原体的干扰，需结合多种检测手段进行综合分析和判断。相比之下，在大多数机构中，CTPA是一种更快速、更容易获得的非侵入性检测手段。本研究例1和例2通过CTPA发现VOS阳性，诊断考虑IPA，予积极抗真菌治疗后，病灶明显缩小。例3通过CTPA检测到VOS阴性的病灶，基本排除IPA，及时停用两性霉素B等抗真菌治疗，随后通过病灶的组织病理活检未发现真菌证据，例3最终确诊为弥漫大B细胞淋巴瘤肺部累及，予抗淋巴瘤治疗后，病情好转，避免了数周不必要的两性霉素B的治疗，减少了真菌耐药发生概率，规避了可能的药物毒性，这与文献报道一致[4],[17]。

尽管在诊断免疫功能低下患者IPA时，CTPA发现的VOS优于经典CT征象，但是关于血管阻塞的现象是曲霉菌病所特有的，还是IPA的一般征兆，目前还无法揭示。在符合EORTC/MSG定义的5例确诊病例中，Stanzani等[15]的队列中包括1例确诊的肺毛霉菌病，同时VOS也呈阳性。但在Sonnet等[14]的研究中，1例肺毛霉菌病患者的VOS呈阴性。因而，CTPA对肺毛霉菌病诊断的意义还有待在毛霉菌病发病率较高的中心进一步研究。

我们承认本研究存在一些局限性。主要是纳入病例数量少，可能存在一些偏倚。CTPA作为检测免疫功能低下患者感染的常规影像学工具也存在一些局限性。首先，由于呼吸运动伪影或技术问题，例如微小结节（小于10 mm）或病灶位于肺底端或肺尖端，CTPA检查可能无法读取。其次，CTPA涉及碘化造影剂的使用，对有肾功能障碍的患者，须谨慎检查，而对无肾脏相关疾病患者，目前无证据证明CTPA会增加肾衰风险[18]。最后，虽然CTPA与肺栓塞的相关检查操作方式相似，但在图像重建和解读方面仍需具备相关的专业知识，可能会面临专业技术人员短缺的问题。

综上，我们报道的病例资料及既往文献资料均表明，CTPA识别的VOS是血液恶性肿瘤患者诊断IPA的一个比较敏感特异的影像学征象。VOS的识别有助于提高IPA诊断的准确性和及时性，帮助指导治疗决策。
